# Immune activation by combination human lymphokine-activated killer and dendritic cell therapy

**DOI:** 10.1038/bjc.2011.290

**Published:** 2011-08-16

**Authors:** E J West, K J Scott, V A Jennings, A A Melcher

**Affiliations:** 1 Cancer Research UK Clinical Centre, St James's University Hospital, Beckett Street, Leeds LS9 7TF, UK

**Keywords:** dendritic cells, lymphokine-activated killer cells, natural killer cells, melanoma, immunotherapy

## Abstract

**Background:**

Optimal cellular immunotherapy for cancer should ideally harness both the innate and adaptive arms of the immune response. Lymphokine-activated killer cells (LAKs) can trigger early innate killing of tumour targets, whereas long-term adaptive-specific tumour control requires priming of CD8+ cytotoxic lymphocytes (CTLs) following acquisition of tumour-associated antigens (TAAs) by antigen-presenting cells such as dendritic cells (DCs). As DCs stimulate both innate and adaptive effectors, combination cell therapy using LAKs and DCs has the potential to maximise anti-tumour immune priming.

**Methods:**

Reciprocal activation between human clinical grade LAKs and DCs on co-culture, and its immune consequences, was monitored by cell phenotype, cytokine release and priming of both innate and adaptive cytotoxicity against melanoma targets.

**Results:**

Co-culture of DCs and LAKs led to phenotypic activation of natural killer (NK) cells within the LAK population, which was associated with increased production of inflammatory cytokines and enhanced innate cytotoxicity against tumour cell targets. The LAKs reciprocally matured DCs, and the combination of LAKs and DCs, on addition of melanoma cells, supported priming of specific anti-tumour CTLs better than DCs alone.

**Conclusion:**

Clinical-grade LAKs/DCs represents a practical, effective combination cell immunotherapy for stimulation of both innate and adaptive anti-tumour immunity in cancer patients.

Induction of efficient immunity against tumours requires a coordinated interplay between the innate and adaptive arms of the immune response. Dendritic cells (DCs) are components of the early innate immune system and can also prime specific, adaptive responses. In particular, cross-presentation of tumour-associated antigens (TAAs) via MHC-I molecules on the surface of DCs can expand specific cytotoxic lymphocytes (CTLs), potentially leading to a long-term anti-tumour memory response (Banchereau and Steinman, 1998).

Natural killer (NK) cells are early innate immune effectors that can exert direct nonspecific cytotoxicity against tumour cells (Srivastava *et al*, 2008). In addition to their role as initiators of antigen-specific responses, DCs also support the tumouricidal activity of NK cells (Fernandez *et al*, 1999), and the extent of cross-talk between DCs and NK cells, leading to reciprocal activation of both cell subsets, has become increasingly recognised (Kalinski *et al*, 2005; Semino *et al*, 2005). Significantly, NK/DC interactions can promote the generation of tumour-specific T-cell responses, whereby NK cells function as nominal ‘helper’ cells to support adaptive anti-tumour immunity (Reschner *et al*, 2008).

Lymphokine-activated killer cells (LAKs) are a heterogeneous population of cells consisting primarily of NK, NKT and T cells, which are generated *in vitro* by culture of peripheral blood mononuclear cells (PBMCs) in IL-2 (Grimm *et al*, 1982). The predominant effector cells within LAKs are NK cells, which are mechanistically equivalent to peripheral blood NK cells, but are more cytotoxic against tumour cells, including otherwise NK-resistant targets (Grimm *et al*, 1982). Moreover, LAKs are more readily cultured in large numbers for administration to patients than purified NK cells. LAKs have previously been shown to localise to tumour sites in both mouse (Kjaergaard *et al*, 2000) and human systems (Keilholz *et al*, 1994), and therefore have the potential to access and lyse tumours in patients following systemic administration *in vivo*. Although there was considerable clinical interest in LAKs for cancer therapy towards the end of the last century, their application for patients has not progressed, in part due to concerns about the toxicity associated with IL-2, which had to be co-administered to maintain LAK activation *in vivo* (Rosenberg, 1988).

As with NK cells, there is evidence that DCs can reciprocally activate LAKs *in vitro* (Valteau-Couanet *et al*, 2002; Capobianco *et al*, 2006). A recent murine study has also shown that co-injection of LAKs and DCs into tumours led to regression associated with protection against secondary rechallenge (Capobianco *et al*, 2008). These data suggest that combination LAK/DC therapy holds promise as a treatment for cancer, based on the hypothesis that DC-activated LAKs will effectively kill tumour targets to liberate TAAs for uptake by DCs within an inflammatory tumour microenvironment. The TAA-loaded mature DCs will then migrate to secondary lymphoid tissue and present TAAs to resident T cells for priming of an additional adaptive antigen-specific response. Moreover, activation of LAKs by co-administered DCs may remove the need for IL-2 and hence reduce the toxicity associated with LAK therapy in the past.

This preclinical study investigated the interaction between clinically relevant LAKs and DCs, their ability to reciprocally activate each other, and the potential of the LAK/DC combination to prime both innate and adaptive immune responses against melanoma in human *in vitro* priming assays. We show that DCs are effectively matured by LAKs, while maintaining their phagocytic function for effective uptake of potential TAAs. In parallel, LAK cytotoxicity is enhanced by co-culture with DCs, as is secretion of inflammatory IFN*γ* and TNF*α*. Furthermore, the addition of LAKs to tumour cell co-cultures with DCs increases specific CTL priming. Hence, LAKs/DCs have potential as a combination cell immunotherapy for priming of both innate and adaptive anti-tumour immunity.

## Materials and methods

### Dendritic cell culture

Buffy coats obtained from healthy donors or whole blood taken from melanoma patients (with written, informed consent in accordance with local institutional ethics review and approval) were used to isolate PBMCs by Ficoll-Hypaque density centrifugation. Monocytes were isolated by MACS CD14+ selection (Miltenyi, Bergisch Gladbach, Germany) according to manufacturer's instructions and were consistently found to be >95% pure. The CD14+ cells were additionally assessed for NK, NKT and T-cell contamination, and were found to be low (average 2.6%, 1.5% and 4.8%, respectively). Immature DCs were generated from CD14+ monocytes in serum-free DC media (CellGro DC media; Cell Genix, Freiberg, Germany) supplemented with 800 IU ml^−1^ GMCSF (Peprotech, London, UK) and 500 IU ml^−1^ IL-4 (R&D Systems, Abingdon, UK) for 5 days. OK432 (Chugai Pharmaceutical Co., Tokyo, Japan) was used at 1000 IU ml^−1^ to generate mature DCs (West *et al*, 2009).

### Lymphokine-activated killer cell generation

CD14− PBMC, isolated following CD14 selection (as above), were routinely found to be negative for CD14+ cell contamination (<5%). LAKs were generated from CD14− PBMC in LAK media (CellGro SCGM media; Cell Genix) supplemented with 5% (v/v) pooled human serum (Sera Labs, Haywards Heath, UK) and 1000 IU ml^−1^ IL-2 (Peprotech) for 5–7 days in non-coated tissue culture flasks (Berdeja *et al*, 2007).

### Tumour cell lines

K562, Daudi and Skov3 cells were maintained in RPMI-1640 (Sigma, Gillingham, UK) supplemented with 10% (v/v) FCS (BioSera, Ringmer, UK) and 1% (v/v) L-glutamine (Sigma). Melanoma cell lines (Mel888, Mel624, MeWo and SKMel-28) were maintained in DMEM (Sigma) supplemented with 10% (v/v) FCS and 1% (v/v) L-glutamine. All cell lines were routinely tested for *mycoplasma* and found to be free of infection.

### Lymphokine-activated killer cell/DC co-cultures

Freshly harvested LAKs and DCs were co-cultured together at a ratio of 10 : 1, in mixed culture media (50 : 50 LAK:DC media without cytokines) at a density of 2 × 10^6^ ml^−1^ LAKs for 48 h. Tumour cell lines were included at a 1 : 1 ratio with DCs at the onset of culture, as required.

### Flow cytometry

DCs and LAKs were analysed using the following antibodies with appropriate isotype controls. *CD11c+ DCs*: anti-human CD14-PE, CD40-PE, CD83-PE, CD86-PE, HLA-DR-PE, MICA/B-PE, CD155-PE, MHC class 1-PE, ICAM-1-PE (BD Biosciences, Oxford, UK), CCR7-PE, ULBP1-PE, ULBP2-PE, CD112-PE, CCR1-PE, CCR5-PE, CXCR1-PE and CXCR2-PE (R&D Systems). LAK (*CD3+CD56−, CD3-CD56+* and *CD3+CD56+*): anti-human CD3-PerCP, CD56-FITC, CD8-FITC, CD16-FITC, CD69-FITC, NKG2D-PE, CCR7-PE, DNAM-1-PE, NKp30-PE, NKp44-PE, LFA-1(CD18)-PE, CD40 L(CD154)-PE (BD Biosciences), CD56-PE (Serotec, Oxford, UK) and NKp46-PE (Miltenyi). Flow cytometry was performed using a BD FACSCalibur and analysed using BD CellQuest Pro software.

### ELISA

Supernatants from 48 h LAK/DC co-cultures were assessed for levels of IL-4, IL-5, IL-6, IL-8, IL-10, IL-12 (p40 and p70), TNF*α* and IFN*γ* by ELISA using antibody matched pairs (all from BD Biosciences except TNF*α*, which was obtained from Invitrogen, Paisley, UK) according to the manufacturer's instructions.

### Degranulation assay

LAK degranulation as a response to target recognition was measured using CD107 surface expression as previously described (Prestwich *et al*, 2008). Briefly, LAKs were co-cultured±DCs for 48 h before co-incubation with tumour targets (1 : 1 ratio LAK:tumour cell) for 4 h, before CD107 staining. LAKs were additionally stained for CD3 and CD56. Analysis was performed by flow cytometry.

### Cytotoxicity assay

DC and LAK cytotoxicity was measured using a standard 4 h ^51^Cr release assay as previously described (Errington *et al*, 2006). Briefly, ^51^Cr-labelled cell targets were incubated with LAKs, DCs or combination LAKs/DCs (pre-cultured together for 48 h) at varying effector to target (E:T) ratios. To examine the perforin dependence of LAK cytotoxicity, E:T co-cultures were set up±2 mM EGTA (Sigma). Percent lysis was calculated using the following formula: % lysis=100 × (sample c.p.m.−spontaneous c.p.m.)/(maximum c.p.m.−spontaneous c.p.m.).

### Dendritic cell phagocytosis assay

DCs were pulsed with FITC-dextran (Sigma)±LAKs (1 : 10 ratio) for 60 min at 37°C (or 4°C control). DCs were harvested at 15 min intervals, stained with CD11c-PE and then analysed by flow cytometry for FITC-dextran uptake.

### Generation of tumour-specific CTL

DCs were cultured with Mel888 cells for 48 h, either in the presence or absence of LAKs, at 1 : 3 : 10 ratio, respectively, to permit loading of antigen on to the DCs, as previously described (Errington *et al*, 2006). These tumour-loaded DCs were then mixed with autologous PBMCs at a 1 : 30 ratio to generate tumour-specific CTLs. CTLs were re-stimulated 7 days later with a second population of tumour-loaded DCs±LAKs. At 14 days, CTLs were harvested and used immediately in cytotoxicity studies against Mel888 and an irrelevant target (Skov3).

### Statistical analyses

Statistical analysis was performed using paired *t*-tests where *P*<0.05 denotes a significant result.

## Results

### Lymphokine-activated killer cells are a heterogeneous population consisting of NK, NKT and T cells

**Figure 1:**
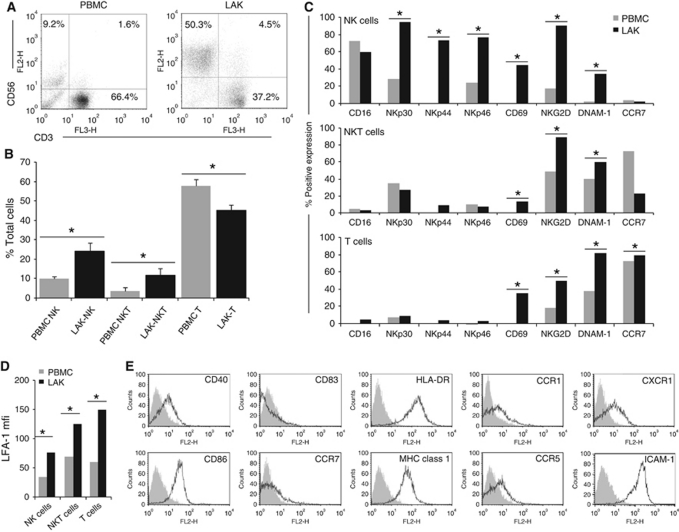
Phenotype of clinical grade LAKs and DCs. Phenotype analysis was performed on CD14− PBMCs before and after culture for 5 days in IL-2 to generate LAKs. (**A**) Relative populations of NK (CD3− CD56+), NKT (CD3+CD56+) and T cells (CD3+CD56−) are shown as representative plots from one donor (of *n*=10); (**B**) mean % of total cells (PBMCs grey bars, LAKs black bars)±s.e.m (of *n*=10 donors; ^*^P<0.05). Expression of activation markers on LAKs are shown as representative plots from one donor (of *n*=5): (**C**) % positive expression and (**D**) mean fluorescence intensity of LFA-1 (^*^P<0.05). DCs were generated by culture of CD14+ PBMCs with GMCSF and IL-4 for 5 days. (**E**) Phenotype analysis is shown as representative FACS plots from one donor (of *n*=5; DCs: black; isotype controls: grey).

This preclinical study used Good Manufacturing Practice-compliant components to generate both LAKs and DCs, to ensure translational relevance for application in human clinical trials. Initial phenotypic analysis of LAKs alone revealed a mixed population consisting predominantly of CD3-CD56+ cells (NK-LAK), CD3+CD56+ cells (NKT-LAK) and CD3+CD56− cells (T-LAK). The relative proportions of the three sub-populations significantly changed during LAK generation by culture of PBMCs in IL-2 (Figure 1A and B). The NK cells increased from 10.1±1.2% in PBMCs to 24.2±4.1% in LAK, NKT cells from 3.9±1.9% to 11.7±3.5%, whereas the T-cell population reduced from 57.7±3.6% to 45.3±2.7%.

Investigations into changes in expression of cell surface molecules during LAK cell generation were performed on these three predominant cell types (Figure 1C and D). Cells of interest were initially identified by their CD3/CD56 expression, as designated in Figure 1A, before analysis of specific activation markers. A significant increase in expression of both NK-specific activation markers (NKp30, NKp44 and NKp46) and general activation markers (CD69, NKG2D and DNAM-1) in IL-2-activated NK-LAK, in comparison with resting NK cells in PBMCs, was observed. In agreement with other studies, NKp30 and NKp46 were detected at low levels on resting NK cells and increased on activation, whereas NKp44 was found only on activated cells (Bryceson *et al*, 2006). The NK-LAK CD16 levels were high at the onset of culture and remained so, whereas CCR7 expression was consistently low. CD40L staining was low or absent on NK-, NKT- and T-LAK (data not shown). The NKT-LAK increased expression of CD69, NKG2D and DNAM-1 compared with NKT in PBMCs, although NK-specific markers remained low or absent (Figure 1C). As expected, the T-cell fraction of PBMCs or LAKs exhibited very low positivity for all NK-specific receptors; however, other activation markers and CCR7 increased following LAK generation (Figure 1C). The adhesion molecule LFA-1 was found on all subsets, and its expression uniformly increased following culture in IL-2 (Figure 1D).

### Clinical grade DCs exhibit an immature phenotype

CD11c-positive clinical DCs were subjected to phenotype analysis of cell surface markers and found to be positive for CD40, CD86, MHC-I and class II (HLA-DR) (Figure 1E), but negative for CD83, CCR7 and CD14 (data not shown): a phenotype indicative of immature DCs (West *et al*, 2009). Other markers, including ligands for activating NK receptors (MICA/B, ULBP1 and 2, CD112), were not expressed (data not shown). ICAM-1 was highly expressed (Figure 1E), correlating with high expression of its receptor, LFA-1, on LAK (Figure 1D). Staining for chemokine/cytokine receptors (CCR1, CCR5 and CXCR1) was also positive (Figure 1E).

### Dendritic cells activate LAKs

**Figure 2:**
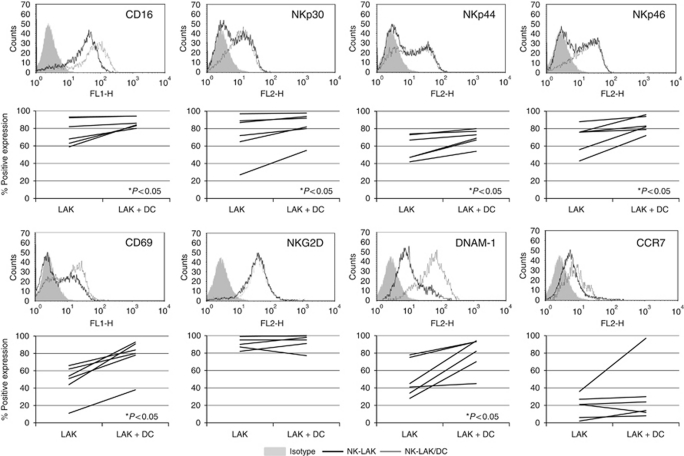
DCs mediated activation of NK-LAK. LAKs and DCs were co-cultured at 10 : 1 ratio for 48 h before NK-LAK (CD3-CD56+) phenotype analysis. Upper panels: representative histograms from one donor showing expression of cell surface markers on NK-LAK cultured in the absence (black) or presence (grey) of DCs; isotype controls are shaded. Lower panels: Line graphs denoting data from all six donors tested (^*^P<0.05).

Experiments were performed to explore potential reciprocal activation between clinical grade LAKs and DCs. Expression of NK-LAK cell surface markers is shown in Figure 2. There was significant upregulation of CD16, NKp30, NKp44 and NKp46 on NK-LAK when co-cultured with DCs for 48 h. Stimulation of the early activation marker CD69 was also apparent, as was upregulation of DNAM-1. However, levels of both NKG2D (already highly expressed) and CCR7 (consistently low) on NK-LAK were not significantly altered by DCs. A similar trend of DC-induced activation was also seen on NKT-LAK and T-LAK (for non-NK specific markers only), although this did not reach statistical significance (Supplementary Figure 1). The LFA-1 on NK-LAK was further enhanced by DCs (data not shown).

### Lymphokine-activated killer cells induce DC maturation

**Figure 3:**
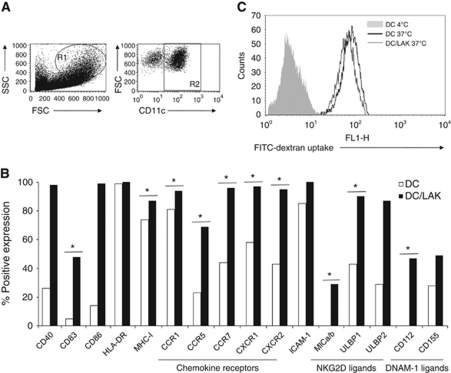
DCs mature in the presence of LAKs, but retain their phagocytic ability. The DCs and LAKs were co-cultured at 1 : 10 ratio for 48 h. (**A**) Representative plots show sequential gating strategy for CD11c+ DCs. (**B**) Representative plot from one donor showing % expression of DC surface markers cultured in the absence (white bars) and presence (black bars) of LAKs (^*^P<0.05 across six donors). (**C**) Uptake of FITC-dextran by DCs alone at 4°C (shaded) or 37°C (black), or in the presence of LAKs at 37°C (grey) during 60 min incubation (representative of four donors).

We next addressed the effects of LAKs on DCs. Although previous work has shown that DCs can be lysed by autologous NK/LAK cells (Parajuli *et al*, 1999), there was no significant killing of DCs by LAK in our clinical grade system (Supplementary Figure 2). Analogous to DC-induced LAK activation, LAKs effectively matured autologous DCs on co-culture (Figure 3B). Maturation was apparent from significant upregulation of CD83, CCR7 and MHC-I expression. Enhancement of CD40 and CD86 was also seen, although this did not reach statistical significance over all six donors. An increase in the levels of the cytokine/chemokine receptors CCR1, CCR5, CCR7, CXCR1 and CXCR2 on DCs in the presence of LAKs was also seen, as was upregulation of the activating NK receptor ligands, MICA/B, ULBP1 and ULBP2 (ligands for NKG2D), as well as CD112 and CD155 (ligands for DNAM-1). In addition, we observed some increase in the already high levels of ICAM-1, although this was donor dependent (data not shown).

To assess this combination therapy within a clinical setting, we performed additional experiments with blood taken from melanoma patients with metastatic disease (stage IV), not on active current treatment. The data demonstrate that LAKs and DCs can be successfully generated from patient PBMCs, and that they similarly cross-activate each other, as demonstrated by enhanced expression of cell surface activation/maturation molecules (Supplementary Figure 3).

We also tested the phagocytic capacity of DCs co-cultured with LAKs, to determine whether LAK-matured DCs retained the ability to take up TAAs potentially released by LAK-lysed tumour cells. For this, uptake of FITC-dextran by DCs was measured±LAK. Figure 3C demonstrates that, even on co-culture with maturation-inducing LAKs, DCs remained functional for uptake of exogenous material, an essential step for DC-mediated priming of an adaptive anti-tumour CTL response.

### Reciprocally activated LAKs/DCs secrete inflammatory cytokines

**Figure 4:**
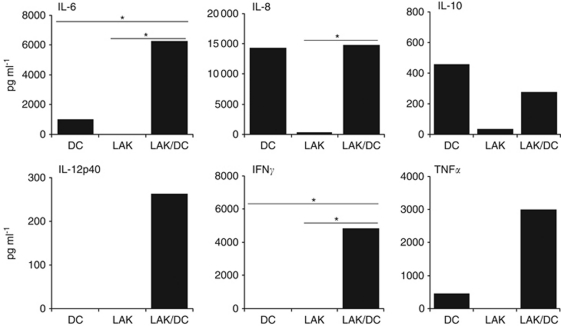
Cytokine secretion by LAK/DC co-cultures. Levels of cytokines were measured in supernatants from 48 h cultures of LAKs, DCs or LAKs/DCs (10 : 1 ratio) by ELISA. Plots show one representative donor (^*^P<0.05 across six donors).

The cytokine profile of LAK/DC co-culture supernatants was measured by ELISA (Figure 4). Increases in secretion of the inflammatory cytokines IL-6, IL-8 and IFN*γ* were observed, together with a nonsignificant trend for IL-12p40 and TNF*α*, in LAK/DC co-cultures compared with LAKs and/or DCs alone. However, IL-12p70 remained undetectable (data not shown), whereas anti-inflammatory IL-10 production by DCs showed a trend to reduction on co-culture with LAKs, although this was donor dependent and not statistically significant across multiple donors.

### Melanoma cells do not inhibit LAK/DC reciprocal activation

Melanoma cells are known to suppress the function of immune cells, including DCs and NK cells (Real *et al*, 2001). As we are proposing LAKs/DCs as a cytotoxic and immunogenic combination cell therapy on interaction with tumour cell targets, we next investigated whether the presence of melanoma cells would have a deleterious effect on the cross-activation between LAKs and DCs described above. The LAKs/DCs were therefore co-cultured with the human melanoma cell lines Mel888 or MeWo, which were found to have no significant effect on reciprocal LAK/DC activation, as demonstrated both by cell phenotype data and cytokine production (Supplementary Figures 4 and 5).

### Dendritic cells enhance perforin-dependent LAK cytotoxicity against tumour targets

**Figure 5:**
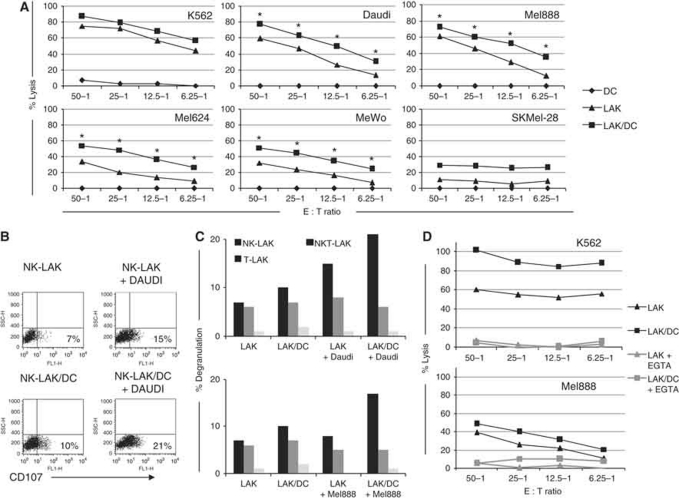
DCs enhance LAK cytotoxicity. LAKs, DCs or LAKs pre-cultured with DCs for 48 h (10 : 1 ratio) were used in: (**A**) ^51^Cr-release assays against targets as shown at various E : T ratios (graphs show one representative donor and asterisks (^*^) denote *P*<0.05 comparing LAKs/DCs *vs* LAKs across five donors); (**B** and **C**) degranulation assays measuring CD107 expression on the surface of LAKs in the absence or presence of DCs and/or tumour targets (representative of *n*=3); (**D**) ^51^Cr-release assays in the absence (black) or presence (grey) of EGTA (representative of *n*=2).

To test whether DC-mediated activation of LAKs enhanced their killing of tumour targets, cytotoxic activity was measured using standard 4 h ^51^Cr-release assays. The LAK, DC and LAK/DC killing of four melanoma cell lines (alongside NK-sensitive K562 and NK-resistant Daudi targets) is shown in Figure 5A. As expected, DCs alone did not kill tumour targets. LAKs exhibited significant cytotoxicity against both K562 and Daudi cells, and were also variably active against the different melanoma lines. Pre-activation of LAKs by DCs resulted in consistent and significantly enhanced killing of the majority of tumour targets (∼20% over that observed for LAKs alone at all E:T ratios). Substantial killing of melanoma targets was also seen using patient-derived LAKs, which was enhanced by pre-incubation with DCs (Supplementary Figure 3).

As ^51^Cr-release assays measure target killing by LAKs as an entire population, to distinguish the relative cytolytic activity of the cell populations within LAKs, degranulation assays were used. The degree of CD107 expression was measured on the surface of LAKs during exposure to Daudi and Mel888, following pre-culture±DCs for 48 h (Figure 5B and C). The results show the NK-LAK fraction degranulated most following co-culture with targets. Although there was measurable background degranulation of both NK-LAK and NKT-LAK in the absence of targets, only CD107 on NK-LAK increased against Daudi and Mel888 following previous activation of LAKs by DCs. T-LAKs did not degranulate under any conditions tested.

To determine the mechanism of cytotoxicity utilised by LAKs, we repeated Cr^51^-release assays in the presence of EGTA (Figure 5D). The reduction in LAK (and LAK/DC) cytotoxicity in the presence of EGTA, which chelates the calcium required for perforin release, is consistent with our previous data demonstrating that DC-stimulated NK cell cytotoxicity is a perforin-dependent process (Errington *et al*, 2008).

### Lymphokine-activated killer cells enhance the ability of DCs to prime an antigen-specific CTL response

**Figure 6:**
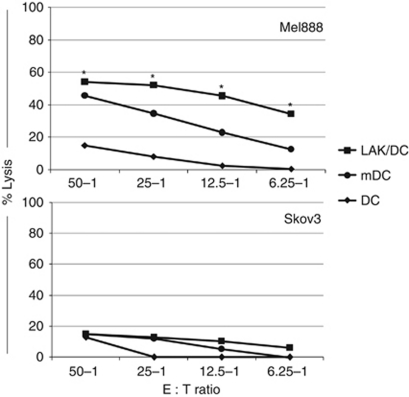
LAKs enhance priming of specific anti-tumour CTLs by DCs. CTLs were generated by culture of DCs±LAKs with Mel888 cells (1 : 10 : 3 ratio) for 48 h before stimulation of PBMC at 1 : 30 ratio for 7 days. The CTLs were restimulated with further tumour cell-loaded DCs±LAKs at day 7, harvested on day 14 and used in 4 h ^51^Cr-release assays against Mel888 or irrelevant (Skov3) targets, at various E : T ratios. OK432-matured tumour cell-loaded DCs were used as a positive priming control (graph shows one representative donor and asterisks (^*^) denote *P*<0.05 comparing LAK/DC *vs* DC priming across five donors).

Data thus far has demonstrated an effective interaction between DCs and LAKs, resulting in reciprocal activation and enhancement of nonspecific innate immune killing of melanoma targets, predominantly by NK-LAK. As DCs form a direct link between innate and adaptive immune responses, we investigated the potential of LAKs/DCs to support adaptive priming of specific CTLs subsequent to early innate tumour killing. Tumour-specific CTLs were generated against Mel888 targets using DCs alone or LAKs/DCs in an MHC class I-dependent *in vitro* human CTLs priming assay (Errington *et al*, 2006). As anticipated, a positive control comprising DCs matured with the bacterial adjuvant OK432 (West *et al*, 2009) induced an adaptive Mel888-specific response in autologous T cells, whereas CTL priming by immature DCs alone was ineffective (Figure 6). However, the combination of LAKs and immature DCs resulted in potent specific CTL priming, with even higher levels of killing than those elicited by OK432-matured DCs. Hence, the ability of co-cultured LAKs to kill targets and/or mature DCs improves adaptive anti-tumour immune priming.

## Discussion

The aim of this preclinical study was to investigate the innate and adaptive immune anti-tumour potential of a deliverable, clinical grade human LAK/DC combination cell therapy for the treatment of cancer, in particular melanoma. LAKs are a clinically applicable heterogeneous population of innate cytotoxic cells consisting predominantly of NK, NKT and T cells. On *ex vivo* culture of human PBMCs in IL-2, we observed an expansion in NK and NKT cells, with a corresponding reduction in T cells (Figure 1B). This is consistent with previously published data (Valteau-Couanet *et al*, 2002; Capobianco *et al*, 2006), suggesting that NK cells are the main effectors within LAKs. The NK-LAKs were activated compared with ‘resting’ NK cells in PBMCs, as indicated by expression of both general surface activation markers (CD69, NKG2D and DNAM-1) and the NK-specific natural cytotoxicity receptors (NCRs) NKp30, NKp44 and NKp46; NKT-LAKs and T-LAKs were also activated by IL-2 (Figure 1C). The DCs used in this study exhibited a typical immature phenotype with low CD83 and CCR7 expression, high MHC-I and II and positive staining for CCR1, CCR5 and CXCR1 (Figure 1E). Notably, ligands for NK activating receptors were initially present at low levels on DCs (Figure 3B), whereas the adhesion molecule ICAM-1 (the ligand for LFA-1 as expressed on LAKs, Figure 1D) was consistently expressed at high levels.

Mouse and human LAK/NK cells are known to activate DCs *in vitro* (Valteau-Couanet *et al*, 2002; Capobianco *et al*, 2006) and vice versa (Kalinski *et al*, 2005; Yano *et al*, 2006), and we found similar reciprocal activation in our clinical grade LAK/DC co-cultures. The DC-mediated LAK activation was evident from further increased expression of NCRs on NK-LAK, and CD69, DNAM-1 and NKG2D on NK-, NKT- and T-LAK (Figure 2). In parallel, LAKs induced maturation of DCs (Figure 3B). Multiple mechanisms are thought to underlie NK/DC cross-talk, including contact-dependent interactions involving CD40/CD40L (Yano *et al*, 2006), NKp30 (Vitale *et al*, 2005) and DNAM-1 (Chan *et al*, 2010) in cooperation with NKp30 (Balsamo *et al*, 2009). Although the detailed interactions between LAKs/DCs are unknown, our data clearly show that following co-culture: (i) NCR on NK-LAK were upregulated (Figure 2) and (ii) known ligands for NK cell activating receptors NKG2D (MICA/B, ULBP1 and ULBP2) and DNAM-1 (CD112 and CD155) were increased on DCs (Figure 3). LFA-1 present on LAKs, alongside ICAM-1 expression on DCs, was also further enhanced during co-culture. LFA-1 and ICAM-1 are involved in cell–cell interaction during an inflammatory response, and LFA-1 is particularly important during NK interaction with antigen-presenting cells and T cells (Borg *et al*, 2004). Taken together, these findings are consistent with LAK/DC co-culture enhancing the effector functions of NK-LAK via direct cellular cross-talk.

In addition to cell–cell contact, cytokines are also proposed to have a role in NK or LAK/DC cross-activation, and may also direct the nature of the innate and adaptive immune responses they elicit *in vivo*. Enhanced levels of IL-6 (Figure 4) detected in LAK/DC co-cultures can stimulate NK and LAK cell proliferation and cytotoxicity (Iho and Shau, 1994) and neutralise regulatory T-cell suppression to facilitate T-cell activation by DCs (Schmidt *et al*, 2004; Detournay *et al*, 2005). Immunosuppressive IL-10 production by DCs was variably inhibited in the presence of LAKs (Figure 4), a finding consistent with previous data (Schmidt *et al*, 2004). Significant secretion of IL-8 by LAK/DC co-cultures (Figure 4) was detected alongside upregulated CXCR1 and 2 expression on DCs (Figure 3B). As a major function of IL-8 is to induce chemotaxis of cells expressing its receptors (CXCR1 and CXCR2) to sites of inflammation in the initial phases of an innate response (Mukaida, 2000), this data supports the potential for LAK/DC-derived IL-8 to sustain additional endogenous effector cell trafficking into targeted tumour sites *in vivo*. Increased IFN*γ* and TNF*α* were also observed in LAK/DC co-cultures (Figure 4). The NK-secreted IFN*γ* is postulated to be essential for DC maturation (Kalinski *et al*, 2005; Capobianco *et al*, 2006), whereas both IFN*γ* and TNF*α* contribute to induction of stable type-1 polarised DC (so-called ‘DC1’) (Kalinski *et al*, 2005). Hence, if LAKs mature co-administered and/or endogenous DCs *in vivo* in the context of IFN*γ*/TNF*α*, these DCs have the potential to migrate to lymph nodes via CCR7-mediated chemotaxis (Figure 3B) to enhance Th1-adaptive priming.

Although LAKs matured DCs (Figure 3B), these DCs could still take up FITC-dextran (Figure 3C), illustrating their competence for acquiring exogenous material from their immediate environment, an important function for cross-priming of TAAs potentially released by dying tumour cells *in vivo*. Moreover, the presence of melanoma cells did not inhibit cross-activation between LAKs and DCs *in vitro* in terms of phenotype and cytokine production (Supplementary Figures 4 and 5), suggesting that LAKs/DCs may be able to reverse immunosuppression to induce an inflammatory microenvironment within tumours, appropriate for induction of therapeutic immune priming.

Consistent with previous reports that DCs can stimulate NK cytotoxicity against tumour targets (Valteau-Couanet *et al*, 2002; Capobianco *et al*, 2006), we found that DCs enhanced innate tumour cell killing by LAKs (Figure 5A). The NK cells were the predominant population within DC-activated LAKs, which degranulated against targets (Figure 5B and C). Moreover, NK-LAK lysed archetypal NK-resistant Daudi cells, as well as classical NK-susceptible targets (K562; Friberg *et al*, 1996), further demonstrating their potency over that of isolated NK cells. Consistent with our previous data on DC-activated NK cells (Errington *et al*, 2008), this killing was perforin-mediated (Figure 5D). Hence, the phenotypic activation of LAKs by DCs (Figure 2) led to enhanced innate cytotoxicity.

In addition to early direct tumour eradication by LAKs/DCs as a combination cell therapy, we wished to address whether the presence of LAKs could enhance the anti-tumour CTL priming efficacy of DCs, thereby forming a link between the innate and adaptive potential of cellular immunotherapy. DCs can modulate the innate responses of NK cells (Kalinski *et al*, 2005), NKT cells (Takahashi *et al*, 2002) and *γδ*T cells (Dieli *et al*, 2004) to potentially connect with an adaptive anti-tumour memory immune response (Capobianco *et al*, 2008). Moreover, DC/NK cell interactions can circumvent the need for CD4+ T cell help during induction of CTLs (Adam *et al*, 2005), further increasing the appeal of adding LAKs to DCs for enhanced adaptive priming. Figure 6 shows, in an established human *in vitro* system for assessing generation of specific anti-melanoma CTLs, that LAKs consistently improved DC-mediated CTL priming to a level greater than that of OK432-matured DCs. Although it is currently unclear whether the benefit of LAKs in this context is due to their tumour-killing capability leading to enhanced antigen release, their ability to mature DCs, or a combination of the two, this data suggests that a LAK/DC combination has the potential to enhance adaptive as well as innate anti-tumour effects in patients *in vivo*.

In summary, this study shows that clinical grade LAKs and DCs can be readily generated *in vitro* in large numbers, both from healthy donors and patients with advanced melanoma, using established methodologies which have already been applied separately and safely in the clinic. As DCs maintain and activate LAKs, the combination could be applied *in vivo* without addition of toxic, systemic IL-2. Functionally, innate direct cytotoxicity of LAKs is improved by DCs, and adaptive CTL priming by DCs is enhanced by the presence of LAKs. This data supports the development of LAKs/DCs as a practical, clinically deliverable combination cellular immunotherapy for the treatment of cancer, and a clinical protocol in melanoma is currently in development.
