# Supplementary figures and images for: Immune activation by combination human lymphokine-activated killer and dendritic cell therapy
Source: Br J Cancer. 2011 Aug 16;105(6):787–95. doi: 10.1038/bjc.2011.290 (PMC3171008; doi:10.1038/bjc.2011.290)

## Slide 1
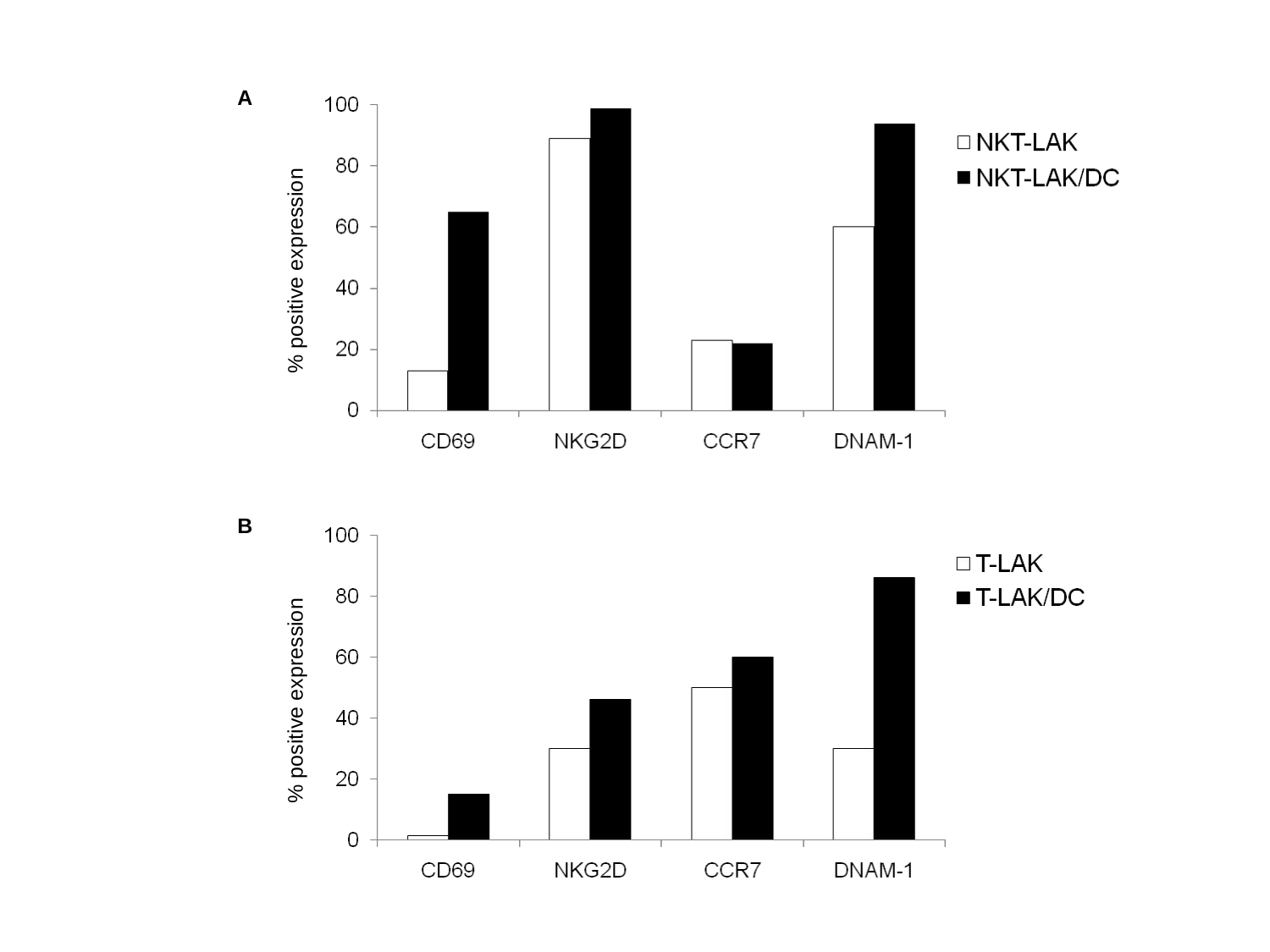

A
% positive expression
B
% positive expression

Supplement: Supplementary Figure 1 [file bjc2011290x1.ppt]

## Slide 1
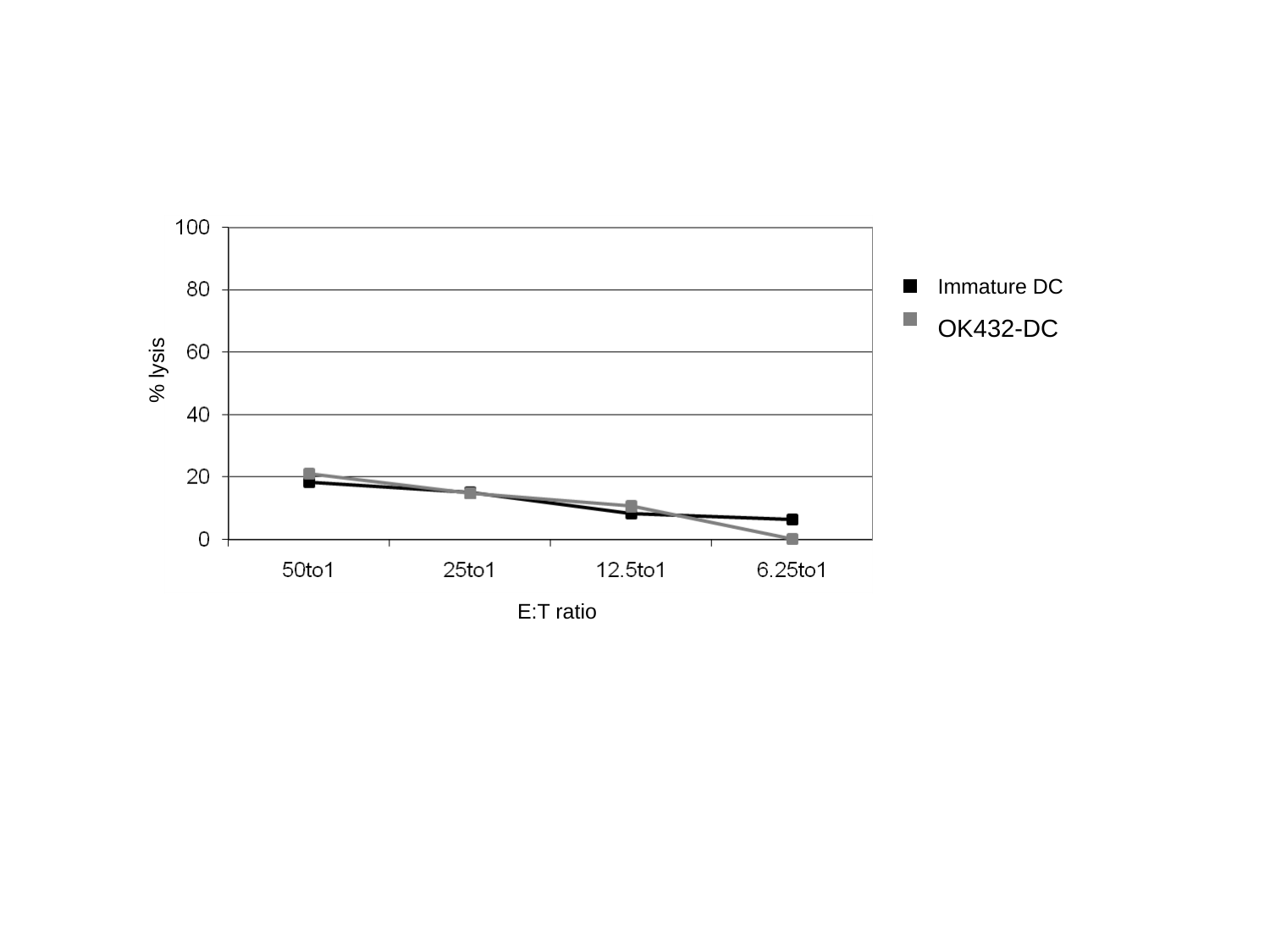

Immature DC
OK432-DC
% lysis
E:T ratio

Supplement: Supplementary Figure 2 [file bjc2011290x2.ppt]

## Slide 1
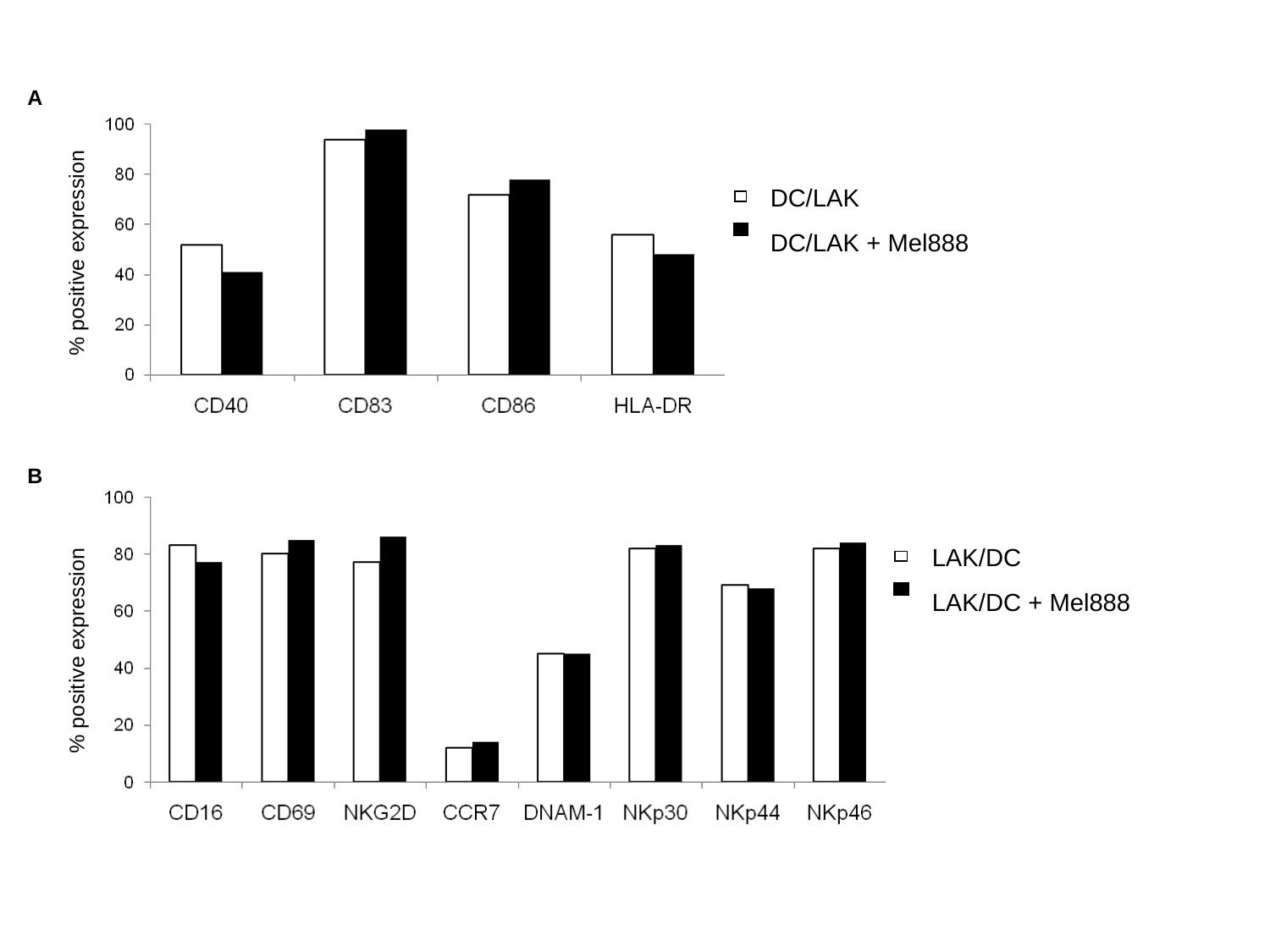

A
DC/LAK
DC/LAK + Mel888
% positive expression
B
LAK/DC
LAK/DC + Mel888
% positive expression

Supplement: Supplementary Figure 4 [file bjc2011290x4.ppt]

## Slide 1
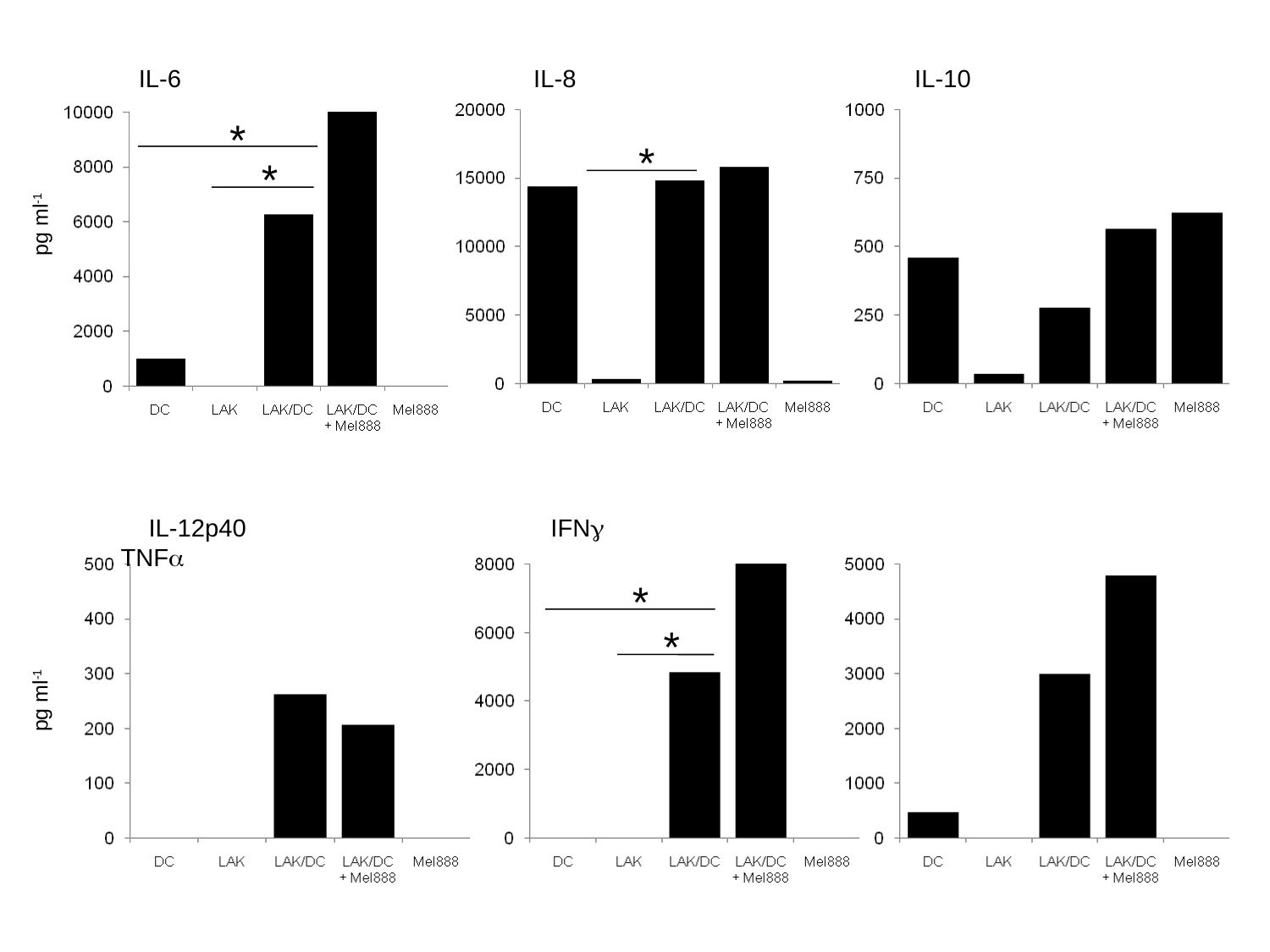

IL-6 	 IL-8	 		 IL-10
*
*
*
pg ml-1
 IL-12p40 	 IFNγ 	 TNFα
*
*
pg ml-1

Supplement: Supplementary Figure 5 [file bjc2011290x5.ppt]
