# Supplementary material for: Immune activation by combination human lymphokine-activated killer and dendritic cell therapy
Source: Br J Cancer. 2011 Aug 16;105(6):787–95. doi: 10.1038/bjc.2011.290 (PMC3171008; doi:10.1038/bjc.2011.290)
Supplement: Supplementary Figure 3 [file bjc2011290x3.ppt]

## Slide 1
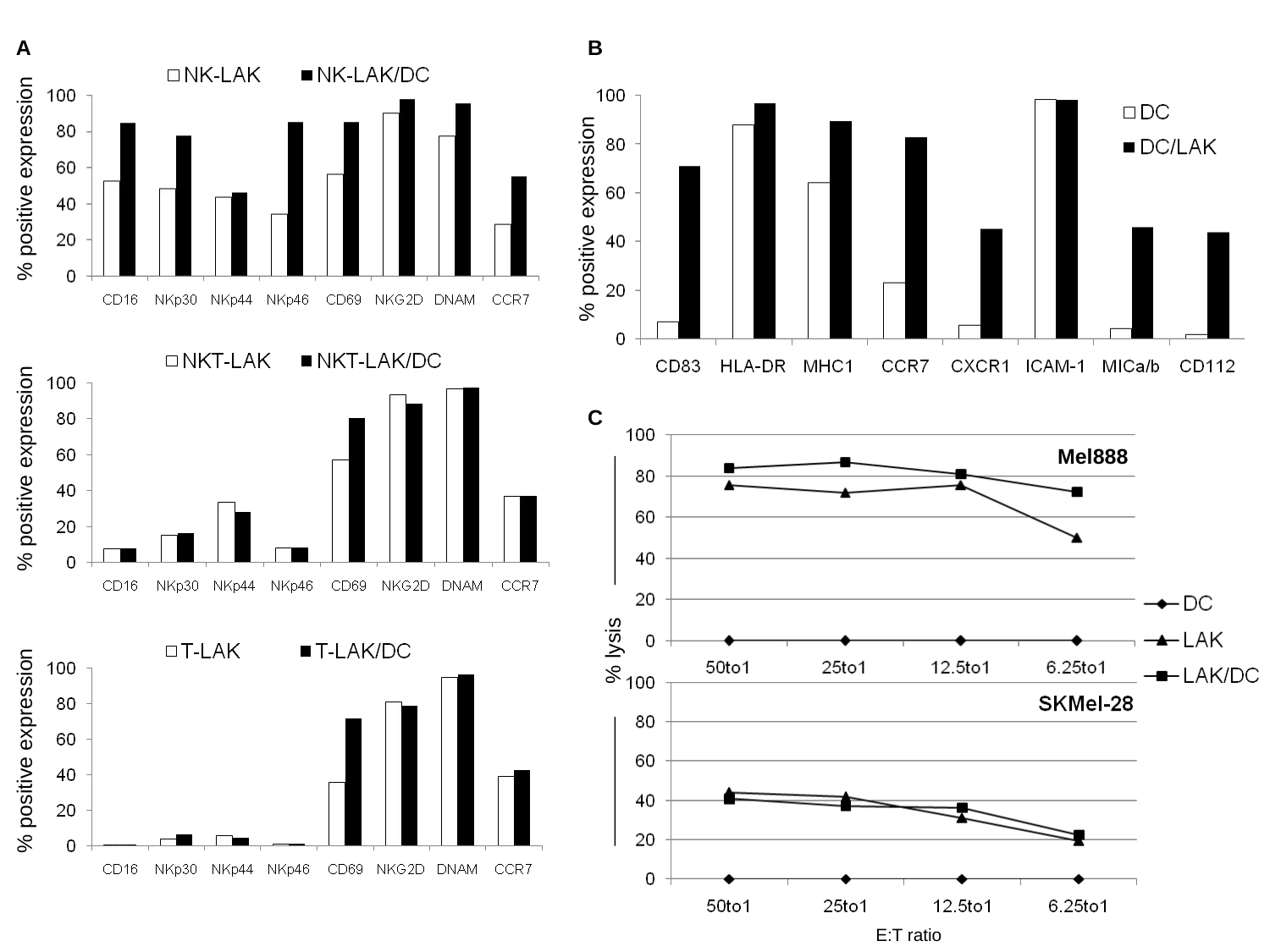

A
B
% positive expression
% positive expression
C
Mel888
% positive expression
% lysis
% positive expression
E:T ratio
