# Supplementary material for: Immune activation by combination human lymphokine-activated killer and dendritic cell therapy
Source: Br J Cancer. 2011 Aug 16;105(6):787–95. doi: 10.1038/bjc.2011.290 (PMC3171008; doi:10.1038/bjc.2011.290)
Supplement: Supplementary Figure Legends [file bjc2011290x6.doc]

**Supplementary Figure Legends**

**Supplementary Figure 1**

*DC-mediated activation of NKT-LAK and T-LAK*.

LAK and DC were co-cultured at 10:1 ratio for 48 hrs prior to phenotype analysis. Representative histograms from 1 donor (of n=4) showing expression of cell surface markers on (A) CD3+CD56+ NKT-LAK or (B) CD3+CD56- T-LAK cultured in the absence (white) or presence (black) of DC.

**Supplementary Figure 2**

*LAK cells do not lyse DC during co-culture.*

Immature DC or OK432-matured DC were labelled with ^51^Cr prior to 48 hr co-culture with LAK at various E:T ratios to determine LAK killing of DC targets. Representative plot of 1 donor (of n=4) showing immature DC (black) and OK432-matured DC (grey) % lysis by LAK.

**Supplementary Figure 3**

*Melanoma patient LAK and DC are comparable to cells generated from healthy donors.*

LAK/DC generated from melanoma patient PBMC were co-cultured (at 10:1 ratio) for 48 hrs prior to cell phenotype analysis of (A) LAK and (B) DC, as for normal donors in Figures 1, 2 and 3. (C) 51Cr-release assays were also performed against melanoma cell lines (Mel888 and SKMel-28) following 48hr co-culture, as in Figure 5. All plots show 1 representative donor of n=3.

**Supplementary Figure 4**

*Melanoma cells do not inhibit LAK/DC reciprocal phenotype activation.*

LAK/DC were cultured in the absence (white) and presence (black) of Mel888 cells (10:1:3 ratio) for 48 hrs prior to cell surface marker analysis of (A) CD11c+ DC and (B) CD3-CD56+ NK-LAK. Representative plots of 1 donor (of n=4).

**Supplementary Figure 5**

*Melanoma cells do not inhibit LAK/DC inflammatory cytokine release.*

LAK/DC were cultured in the absence or presence of Mel888 cells (10:1:3 ratio) for 48 hrs prior to measurement of cytokine secretion by ELISA. Mel888 cells, LAK and DC were cultured alone as controls. Representative plots of 1 donor (*p<0.05 for n=4).
